# ERK phosphorylation disrupts the intramolecular interaction of capicua to promote cytoplasmic translocation of capicua and tumor growth

**DOI:** 10.3389/fmolb.2022.1030725

**Published:** 2022-12-22

**Authors:** Jongmin Park, Guk-Yeol Park, Jongeun Lee, Joonyoung Park, Soeun Kim, Eunjeong Kim, Seung-Yeol Park, Jong Hyuk Yoon, Yoontae Lee

**Affiliations:** ^1^ Department of Life Sciences, Pohang University of Science and Technology, Pohang, Gyeongbuk, South Korea; ^2^ Department of Biology, College of Natural Sciences, Kyungpook National University, Daegu, South Korea; ^3^ Neurodegenerative Diseases Research Group, Korea Brain Research Institute, Daegu, South Korea; ^4^ Institute of Convergence Science, Yonsei University, Seoul, South Korea

**Keywords:** CIC, ERK, receptor tyrosine kinase, cytoplasmic translocation, tumor suppressor

## Abstract

Activation of receptor tyrosine kinase signaling inactivates capicua (CIC), a transcriptional repressor that functions as a tumor suppressor, *via* degradation and/or cytoplasmic translocation. Although CIC is known to be inactivated by phosphorylation, the mechanisms underlying the cytoplasmic translocation of CIC remain poorly understood. Therefore, we aimed to evaluate the roles of extracellular signal-regulated kinase (ERK), p90RSK, and c-SRC in the epidermal growth factor receptor (EGFR) activation-induced cytoplasmic translocation of CIC and further investigated the molecular basis for this process. We found that nuclear ERK induced the cytoplasmic translocation of CIC-S. We identified 12 serine and threonine (S/T) residues within CIC, including S173 and S301 residues that are phosphorylated by p90RSK, which contribute to the cytoplasmic translocation of CIC-S when phosphorylated. The amino-terminal (CIC-S-N) and carboxyl-terminal (CIC-S-C) regions of CIC-S were found to interact with each other to promote their nuclear localization. EGF treatment disrupted the interaction between CIC-S-N and CIC-S-C and induced their cytoplasmic translocation. Alanine substitution for the 12 S/T residues blocked the cytoplasmic translocation of CIC-S and consequently enhanced the tumor suppressor activity of CIC-S. Our study demonstrates that ERK-mediated disruption of intramolecular interaction of CIC is critical for the cytoplasmic translocation of CIC, and suggests that the nuclear retention of CIC may represent a strategy for cancer therapy.

## Introduction

Capicua (CIC) is a transcriptional repressor evolutionarily conserved in several species ranging from *Caenorhabditis elegans* to humans (Jimenez et al., 2000). In mammals, CIC participates in the regulation of various developmental processes, including abdominal wall closure during embryogenesis, lung alveolarization, brain development, and lymphocyte development (Lee et al., 2011; Lu et al., 2017; Park et al., 2017; Simon-Carrasco et al., 2017; Tan et al., 2018; Ahmad et al., 2019; Kim et al., 2021; Hong et al., 2022). CIC additionally regulates the pathogenesis of various diseases, such as spinocerebellar ataxia type-1, autoimmune disease, and liver injury (Fryer et al., 2011; Park et al., 2017; Park et al., 2019; Park et al., 2020). Among these diseases, the role of CIC in cancer has been most extensively studied. Mutations and loss of CIC have been reported to promote the progression of various cancers *via* derepression of cancer-associated CIC target genes, including polyomavirus enhancer activator 3 (*PEA3*) group genes (ETS variant transcription factor 1 [*ETV1*], *ETV4*, and *ETV5*) (Kawamura-Saito et al., 2006; Choi et al., 2015; Okimoto et al., 2017; Kim et al., 2018; Bunda et al., 2019; Lee et al., 2020).

CIC is expressed *via* alternative promoters in two different isoforms, long isoform (CIC-L) and short isoform (CIC-S) (Fryer et al., 2011). CIC-L contains a unique amino (N)-terminal region. Both CIC-L and CIC-S have a high-mobility group (HMG) box and a C1 domain, by which they directly bind to specific octameric DNA sequences, T (G/C)AATG (A/G) (A/G), to repress target gene expression (Shin and Hong, 2014; Fores et al., 2017; Weissmann et al., 2018). Activation of the receptor tyrosine kinase (RTK) pathway inactivates CIC *via* degradation and/or cytoplasmic translocation in *Drosophila* and mammals (Jimenez et al., 2012). Deletion of the C2 motif, which is the mitogen-activated protein kinase (MAPK) docking site of CIC, inhibits RTK activation-induced cytoplasmic translocation and degradation of CIC in *Drosophila* (Astigarraga et al., 2007). Deletion of the extracellular signal-regulated kinase (ERK)-binding site similarly suppresses nuclear degradation of CIC induced by epidermal growth factor receptor (EGFR) activation in humans (Futran et al., 2015; Bunda et al., 2019). Consistent with this observation, the inhibition of MAPK kinase (MEK), an upstream kinase of ERK, increases CIC levels in human cells treated with EGF (Okimoto et al., 2017; Wang et al., 2017; Bunda et al., 2019). However, other studies have shown that EGF treatment does not induce CIC degradation in mammalian cells (Ren et al., 2020; Wong et al., 2020), suggesting that the RTK activation-induced CIC degradation may depend on experimental conditions. Inactivation of MEK and p90RSK, a downstream kinase of ERK, suppresses the cytoplasmic translocation of CIC in HEK293T cells upon EGF or fibroblast growth factor treatment (Ren et al., 2020). Specifically, p90RSK phosphorylates CIC at serine (S) 173 and 301 residues (in the case of human CIC-S) to induce 14-3-3-mediated nuclear export of CIC (Ren et al., 2020). Moreover, EGF-induced proto-oncogene tyrosine-protein kinase Src (c-SRC) activation induces cytoplasmic translocation of CIC *via* phosphorylation of tyrosine (Y) 1,455 residue (in the case of human CIC-S) (Bunda et al., 2020). Although a few amino acid residues of CIC and kinases responsible for the RTK activation-induced cytoplasmic translocation of CIC have been identified in mammals, the mechanism by which phosphorylated CIC is transported into the cytoplasm remains poorly understood. Therefore, we aimed to evaluate ERK, p90RSK, and c-SRC for their contribution to the EGFR activation-induced cytoplasmic translocation of CIC and further investigated the molecular basis for this process. Our study revealed that nuclear ERK mediated the cytoplasmic translocation of CIC-S by disrupting the intramolecular interaction of CIC-S and that nuclear retention of CIC suppressed CIC target gene expression and tumor growth.

## Materials and methods

### Cell culture

HEK293T and MHCC-97H cells were cultured in Dulbecco’s modified Eagle medium (DMEM) (LM001-05; Welgene, Gyeongsan, Republic of Korea) supplemented with 10% fetal bovine serum (FBS) (S001-07, Welgene) and 1% penicillin/streptomycin (15140122; Gibco, Waltham, MA, United States) at 37°C in a 5% CO_2_ incubator. For EGF and chemical treatment experiments, HEK293T cells were cultured in DMEM without FBS for 12 h. Cells were routinely screened and were found to be free of mycoplasma contamination.

### EGF and chemical treatment

To induce RTK signaling, HEK293T cells were treated with 0.1 μg/ml of recombinant human EGF (AF-100-15A; Peprotech, Cranbury, NJ, United States) dissolved in phosphate-buffered saline (PBS) containing 0.2% bovine serum albumin. The conditions for the kinase inhibitor treatment were determined based on previous studies: SCH772984 (S7101, SelleckChem, Houston, TX, United States): 10 μM for 2 h (Martinez et al., 2021), DEL22379 (S7921, SelleckChem): 10 μM for 30 min (Herrero et al., 2015), LJH685 (S7870, SelleckChem): 10 μM for 3 h (Ren et al., 2020), and dasatinib (73082, STEMCELL Technologies, Vancouver, Canada): 200 nM for 1 h (Koreckij et al., 2009). To inhibit the proteasome complex, the cells were treated with 10 μM MG132 (C2211, Sigma–Aldrich, St. Louis, MO, United States) for 6 h.

### Plasmid construction and site-directed mutagenesis

The coding sequence (CDS) of mouse *Cic-S* was amplified using Pfu-X DNA polymerase (SPX16; SolGent, Daejeon, Republic of Korea) and cloned into the p3XFLAG-CMV-10 vector (p3XFLAG-CMV-10-CIC-S^WT^; E7658, Sigma–Aldrich) or MIGR1-GFP vector (MIGR1-FLAG-CIC-S^WT^-GFP; 27490; Addgene, Watertown, MA, United States). pHAGE-FLAG-CIC-S was constructed as described previously (Choi et al., 2015). A series of C-terminally truncated CIC-S mutants (CIC-S^Δ1389−end^, CIC-S^Δ1302−end^, CIC-S^Δ1184−end^, CIC-S^Δ910−end^, and CIC-S^Δ601−end^) were cloned into the p3XFLAG-CMV-10 vector. The N-terminal region of CIC-S containing a FLAG tag at the N-terminus (FLAG-CIC-S-N, residues 1–700) and the C-terminal region of CIC-S containing an HA tag at the C-terminus (CIC-S-C-HA, residue 701 to the end) were cloned into an MIGR1-GFP vector.

p3XFLAG-CMV-10-CIC-S^H1∼5A^, p3XFLAG-CMV-10-CIC-S^C1∼5A^, p3XFLAG-CMV-10-CIC-S^H12A^, p3XFLAG-CMV-10-CIC-S^H345A^, and p3XFLAG-CMV-10-CIC-S^Y1451F^ were generated using the QuickChange II XL Site-Directed Mutagenesis kit (200521; Agilent Technologies, Santa Clara, CA, United States) according to the manufacturer’s protocol. The FLAG-CIC-S^H1∼5A^ fragment (1–680 residues) digested using NotI and BstPI was sub-cloned into the p3XFLAG-CMV-10-CIC-S^C1∼5A^ plasmid to construct the p3XFLAG-CMV-10-CIC-S^CH1∼5A^ plasmid. FLAG-CIC-S^CH1∼5A^ was also cloned into the MIGR1-GFP vector (MIGR1-FLAG-CIC-S^CH1∼5A^-GFP) using the BglII and HpaI restriction enzyme sites.

Kinase overexpression studies were performed using pKH3-human RSK1 (13841, Addgene), pcDNA3 c-SRC (42202, Addgene), pCMV-myc-ERK2-L4A-MER1-fusion (39197, Addgene), and pCMV-myc-ERK2-MER1-fusion (39194, Addgene) plasmids. The CDS of mouse *Erk2* was amplified using Pfu-X DNA polymerase and cloned into the pCK-V5 vector (Han et al., 2004). Constitutively active mutations (ERK2 L73P/S151D) were introduced using the QuickChange II XL Site-Directed Mutagenesis kit according to the manufacturer’s protocol. The same amount of p3XFLAG-CMV-10, pCK-V5, and pKH3 (12555, Addgene) plasmids were used for negative control (NC) transfection. pEGFP-C1 (6084-1; TakaraBio, Kusatsu, Japan) plasmid was used as a control for the transfection efficiency. METAFECTENE PRO (T040; Biontex, Munich, Germany) was used for plasmid transfection. Primers used for cloning and mutagenesis are listed in Supplementary Table S1.

### Generation of *CIC-*KO HEK293T cells using the CRISPR-Cas9 system

A *CIC*-targeting CRISPR-Cas9 plasmid was constructed as previously described (Ran et al., 2013). Briefly, a DNA fragment encoding the single guide RNA targeting exon 4 of *CIC* (sgCIC, 5′-CTC​TAC​CGC​CCG​GAA​AAC​GT-3′) was cloned into the pSpCas9(BB)-2A-GFP vector (48138, Addgene) using the BbsI restriction enzyme site. HEK293T cells were grown to 70% confluence and transfected with pSpCas9(BB)-2A-GFP-sgCIC using FuGENE HD (E2311; Promega, Madison, WI, United States) according to the manufacturer’s instructions. Green fluorescent protein positive (GFP^+^) cells were single cell-sorted into 96-well plates using a MoFlo-XDP cell sorter (Beckman Coulter, Brea, CA, United States). Established cell clones were assayed for CIC expression by western blotting to select *CIC-*KO HEK293T cell clones. Genomic DNA flanking exon 4 of *CIC* was amplified by polymerase chain reaction (PCR), and the PCR products were cloned into a T-blunt vector (SOT02-K020, SolGent). Subsequently, the deleted DNA sequences in exon 4 of *CIC* were identified *via* sequencing.

### Virus production and transduction

To generate viruses expressing ERK-kinase translocation reporter (ERK-KTR), HEK293T cells were co-transfected with pLentiPGK Puro DEST ERK-KTRClover (90227, Addgene), pSPAX2 (12260, Addgene), and pDM2.G (12259, Addgene) using FuGENE HD. Viral supernatants were collected 48 h after transfection and concentrated using a Lenti-X Concentrator (631231, TakaraBio) according to the manufacturer’s protocol. The resuspended pellet was used to infect HEK293T cells, and 10 μg/ml puromycin (A1113803, Gibco) was added to select drug-resistant cells at 48 h post-infection.

To generate retroviruses expressing control, FLAG-CIC-S^WT^, or FLAG-CIC-S^CH1∼5A^, the HEK293T cells were co-transfected with gag/pol (14887, Addgene), pVSVg (8454, Addgene), pAdVAntage (E1711, Promega), and either MIGR1-FLAG-CIC-S^WT^-GFP, MIGR1-FLAG-CIC-S^CH1∼5A^-GFP, or MIGR1-GFP control retroviral vector using FuGENE HD. Viral supernatants were collected 48 h after transfection and used to infect the *CIC-*KO HEK293T cells. Subsequently, GFP^+^ cells were sorted using a MoFlo-XDP cell sorter.

For *in vitro* cell growth and *in vivo* tumor growth assays, lentiviruses expressing FLAG-CIC-S^WT^ and FLAG-CIC-S^CH1∼5A^ were generated *via* the same protocol using the pHAGE-FLAG-CIC-S^WT^ and pHAGE-FLAG-CIC-S^CH1∼5A^ plasmids. Viral supernatants were collected 48 h after transfection and used to infect MHCC-97H cells for three sequential days.

### Western blot analysis

Western blot analysis was performed as previously described (Park et al., 2020). Total protein samples were prepared by lysis in radioimmunoprecipitation assay (RIPA) buffer [50 mM Tris-HCl pH 7.4, 150 mM NaCl, 1 mM phenylmethylsulfonyl fluoride, 1% NP-40, 0.5% sodium deoxycholate, 0.1% sodium dodecyl sulfate (SDS), 1× Complete Protease Inhibitor Cocktail (43229800; Roche, Basel, Switzerland), and 1× phosphatase inhibitor cocktail (4906837001, Roche)]. Nuclear and cytoplasmic protein samples were prepared using NE-PER Nuclear and Cytoplasmic Extraction Reagents (78833; Thermo Fisher Scientific, Waltham, MA, United States) according to the manufacturer’s protocol. Protein concentrations were measured using a bicinchoninic acid assay kit (23225, Thermo Fisher Scientific). Equal amounts of protein were prepared and boiled in sample buffer (250 mM Tris-HCl pH 6.8, 50% glycerol, 10% SDS, 5% β-mercaptoethanol, and 0.1% bromophenol blue) for 5 min. Protein samples were separated using 9% SDS-polyacrylamide gel electrophoresis and transferred onto nitrocellulose membranes (162-0115; BioRad, Hercules, CA, United States). Rabbit polyclonal anti-CIC antibodies were generated as previously described (Kim et al., 2015). The primary antibodies used were as follows: anti-FLAG (1:3000 dilution; F7425, Sigma–Aldrich), anti-lamin A/C (1:3000 dilution; 2032S, Cell Signaling Technology, Danvers, MA, United States), anti-α-tubulin (1:2000 dilution; sc-398103, Santa Cruz Biotechnology, Dallas, TX, United States), anti-ERK (1:3000 dilution; 9102S, Cell Signaling Technology), anti-P-ERK (1:1000 dilution; 4370S, Cell Signaling Technology), anti-glyceraldehyde 3-phosphate dehydrogenase (GAPDH; 1:2000 dilution; sc-32233, Santa Cruz Biotechnology), anti-HA (1:2000 dilution; 3724S, Cell Signaling Technology), anti-ETV4 (1:1000 dilution; 10684-1-AP, Proteintech, Rosemont, IL, United States), anti-ETV5 (1:1000 dilution; 13011-1-AP, Proteintech), anti-lamin B (1:1000 dilution; sc-374015, Santa Cruz Biotechnology), anti-β-actin (1:2000 dilution; sc-47778, Santa Cruz Biotechnology), anti-Myc (1:2000 dilution; 71D10, Cell Signaling Technology), anti-c-SRC (1:1000 dilution; 42202, Santa Cruz Biotechnology), and anti-GFP (1:2000 dilution; sc-8334, Santa Cruz Biotechnology). Proteins were visualized using Clarity Western ECL Substrate (170-5061, BioRad) or SuperSignal West Dura Substrate (34076, Thermo Fisher Scientific). Western blot images were obtained using Image Quant LAS 500 (GE Healthcare Life Sciences, Marlborough, MA, United States). The band intensity was quantified using ImageJ software (v. 1.46r; National Institutes of Health, Bethesda, MD, United States).

### Immunocytochemistry

HEK293T_ERK-KTR cells (4 × 10^4^ cells/ml) were seeded onto 6-well plates containing circular cover glasses (Ф12 mm), and the following day, the cells were transfected with p3XFLAG-CMV-10-FLAG-CIC-S^WT^ or p3XFLAG-CMV-10-FLAG-CIC-S^CH1∼5A^ plasmid for 48 h. Cells were fixed with 4% paraformaldehyde (PFA; P2031, BIOSESANG, Seongnam, Republic of Korea) at room temperature (RT; 22°C–26°C) for 10 min and then incubated with cold methanol at −20°C for 10 min. Cells fixed on the cover glasses were blocked in PBS containing 10% FBS and 0.05% NaN_3_ for 1 h and then incubated with a monoclonal ANTI-FLAG M2 antibody (1:100 dilution; F1804, Sigma–Aldrich) in PBS containing 10% FBS, 0.05% NaN_3_, and 0.2% saponin at 4°C overnight. This was followed by incubation with secondary anti-mouse IgG Alexa Fluor 633 (1:500 dilution; A-21053, CiteAb, Bath, United Kingdom) in PBS containing 10% FBS, 0.05% NaN_3_, and 0.2% saponin at 4°C for 1 h. After staining with 4′,6-diamidino-2-phenylindole (DAPI; F6057, Sigma–Aldrich) for 5 min, the cover glass was washed with PBS containing 10% FBS and 0.05% NaN_3_ and mounted on the slide glass using a Fluoromount-G mounting solution (0100-01, SouthernBiotech, Birmingham, AL, United States). Images of the slides were obtained using an LSM 800 confocal microscope (Carl Zeiss, Oberkochen, Germany) with a 63× oil-immersion lens. Fluorescence intensity quantification was performed using the ImageJ software.

### Proximity ligation assay

HEK293T cells (4 × 10^4^ cells/ml) were seeded onto 6-well plates containing circular cover glasses (Ф12 mm), and the following day, the cells were co-transfected with MIGR1-FLAG-CIC-S-N-GFP and MIGR1-CIC-S-C-HA-GFP plasmids using FuGENE HD for 48 h. Cells were fixed with 4% PFA at RT for 10 min and then permeabilized with 0.1% Triton X-100 (TRX777; BIOPURE, Dasan, Republic of Korea) at RT for 10 min. Proximity ligation assay was performed using the Duolink *In Situ* PLA Probe Anti-Rabbit PLUS (DUO92002, Sigma–Aldrich) according to the manufacturer’s protocol. The monoclonal ANTI-FLAG M2 antibody (1:200 dilution) and anti-HA antibody (1:200 dilution) were used as primary antibodies. Images of the slides were obtained using an LSM 800 confocal microscope with a 63× oil-immersion lens.

### Quantitative real-time PCR (qRT-PCR)

Total RNA was extracted using TRIzol reagent (10296-010, Thermo Fisher Scientific), and reverse transcription was performed using the GoScript™ Reverse Transcription System (A5004, Promega) according to the manufacturer’s instructions. SYBR Green real-time PCR master mix (TOQPK-201; Toyobo, Osaka, Japan) was used for the qRT-PCR analysis. The expression of each target gene was analyzed using StepOnePlus™ Real-Time PCR System (Applied Biosystems, Waltham, MA, United States). Expression data were calculated using the 2^−ΔΔCt^ method and presented as relative mRNA expression levels. Gene expression levels were normalized to those of *GAPDH*. The primers used for qRT-PCR analysis were as follows: *ETV5* forward: 5′-CAT​CCT​ACA​TGA​GAG​GGG​GTT​A-3′ and reverse: 5′-AAG​TAT​AAT​GGG​GGA​TCT​TTT​TCA-3′; *DUSP6* forward: 5′-GAA​CTG​TGG​TGT​CTT​GGT​ACA​TT-3′ and reverse: 5′-GTT​CAT​CGA​CAG​ATT​GAG​CTT​CT-3′; *GAPDH* forward: 5′-ACA​ACT​TTG​GTA​TCG​TGG​AAG​G-3′ and reverse: 5′-GCC​ATC​ACG​CCA​CAG​TTT​C-3′.

### Co-immunoprecipitation

To investigate the interaction between the N-terminal and C-terminal regions of CIC-S, HEK293T cells were transfected with FLAG-CIC-S-N and CIC-S-C-HA expression plasmids. The cells were harvested, centrifuged at 500 × g for 5 min, and fixed with 1% PFA in PBS with shaking at 50 rpm at RT for 10 min. The fixation step was terminated by adding 1/20 volume of 2.5 M glycine. After an additional 5 min of incubation with shaking at 50 rpm at RT, fixed cells were collected by centrifugation at 500 *g* for 5 min and washed with PBS. The cell pellets were resuspended in 200 μl of IP buffer (20 mM Tris-HCl pH 8.0, 350 mM KCl, and 0.2 mM EDTA) for 12 cycles of sonication (cycle: 0.5 and amplitude: 50; UP 400 s, Hielscher, Teltow, Germany). After centrifugation at 17,000 × *g* for 15 min, the supernatants were collected and incubated with Protein G Agarose (16-266, Merck, Kenilworth, NJ, United States) for 1 h at 4°C with rotation at 12 rpm for pre-clearing. After centrifugation, the supernatants were incubated with ANTI-FLAG M2 affinity gel (A2220, Sigma–Aldrich) overnight at 4°C. Beads were collected by centrifugation and washed with IP buffer. To elute the immunoprecipitated proteins, the beads were boiled in the sample buffer for 10 min. Eluted proteins were subjected to western blot analysis.

### DNA binding assay

HEK293T cells (4.4 × 10^6^ cells/6 ml) were seeded onto Ф100 mm dish, and the following day, the cells were transfected with p3XFLAG-CMV-10 or p3XFLAG-CMV-10-CIC-S^WT^ expression plasmids. After approximately 72 h, cells were treated with either PBS or EGF for 5min, harvested, and centrifuged at 1300 × g for 5 min. The cell pellets were resuspended in 400 μl of 100 mM KCl IP buffer (20 mM Tris-HCl pH 8.0, 100 mM KCl, 1× Complete Protease Inhibitor Cocktail, and 1× phosphatase inhibitor cocktail) for 16 cycles of sonication (cycle: 0.5 and amplitude: 50). After centrifugation at 17,000 × g for 15 min, protein concentrations were measured using a bicinchoninic acid assay kit. The same amount of proteins was incubated with 4 × 10^–1^ pmole of double stranded DNA (dsDNA) oligomer composed of six consecutive CIC binding motifs in 400 ul of 100 mM KCl IP buffer for 1 h at 37°C. Each sample was incubated with 10 μl of Protein G Agarose for 1 h at 4°C with rotation at 12 rpm for pre-clearing. After centrifugation at 1,000 × g for 2 min, the supernatants were incubated with 10 μl of ANTI-FLAG M2 affinity gel overnight at 4°C. Beads were collected by centrifugation and washed five times with 150 mM KCl IP buffer (20 mM Tris-HCl pH 8.0, 150 mM KCl, 0.2 mM EDTA) for 5 min at 4°C with rotation at 12 rpm. To elute DNA bound to FLAG-CIC-S, the beads were incubated with 100 ul of elution buffer (100 mM NaHCO_3_ and 0.5% SDS) twice for 30 min at RT with rotation at 12 rpm. Approximately 200 ul of the collected eluate was treated with Proteinase K (P1048, BIOSESANG) at 45°C for 1.5 h, and then subjected to DNA purification using Expin™ CleanUp SV (113-102, GeneAll, Seoul, Republic of Korea). Purified DNA was subjected to qPCR analysis. To calculate the amount of immunoprecipitated dsDNA oligomer, Ct values for different amount of dsDNA oligomers (10^–2^, 10^–3^, 10^–4^, 10^–5^, 10^–6^, and 10^–7^ pmole) were also determined by qPCR analysis. The sequences of dsDNA oligomer were as follows: upper strand; 5′-GAC​AAC​TTT​GGT​ATC​GTG​GAA​GGT​GAA​TGA​ATG​AAT​GGA​TGA​ATG​AAT​GAA​TGG​ATG​AAT​GAA​TGA​ATG​GAA​ACT​GTG​GCG​TGA​TGG​CG-3′, and lower strand; 5′-CGC​CAT​CAC​GCC​ACA​GTT​TCC​ATT​CAT​TCA​TTC​ATC​CAT​TCA​TTC​ATT​CAT​CCA​TTC​ATT​CAT​TCA​CCT​TCC​ACG​ATA​CCA​AAG​TTG​TC-3′. The primers used for qRT-PCR analysis were as follows: forward primer; 5′-AAC​TTT​GGT​ATC​GTG​GAA​GGT​G-3′, and reverse primer; 5′-GCC​ATC​ACG​CCA​CAG​TTT​C-3′.

### Cell growth assay

Mock, FLAG-CIC-S^WT^-, and FLAG-CIC-S^CH1∼5A^-expressing MHCC-97H cells (2 × 10^4^ cells/500 μl) were seeded in 24-well plates. Cells were trypsinized and stained with Trypan Blue (T8154, Sigma–Aldrich), and the number of viable cells was counted using a hemacytometer every day for 4 days.

### *In vivo* tumor growth assay

Five-week-old male BALB/c nude mice were purchased from Orient Bio (Seongnam, Republic of Korea). Mice were acclimatized for 1 week and then used for *in vivo* tumor growth assays. Mice were fed standard rodent chow and water *ad libitum* and maintained in a specific pathogen-free animal facility under a standard 12 h light/12 h dark cycle. All experimental procedures were performed in accordance with guidelines and regulations approved by the Institutional Animal Care and Use Committee of POSTECH (POSTECH-2021-0094).

Mock, FLAG-CIC-S^WT^-, and FLAG-CIC-S^CH1∼5A^-expressing MHCC-97H cells were trypsinized, collected, and suspended in DMEM and Matrigel (354234; BD Biosciences, Franklin Lakes, NJ, United States) (1:1 volume). The cells (5 × 10^6^ cells/100 μl) were injected subcutaneously into the posterior flank of six-week-old male BALB/c nude mice. Tumor size was measured every 3 days for 18 days. The tumor volume was calculated as 0.5 × (largest diameter) × (smallest diameter)^2^. Mice were euthanized on day 18, and the tumors were collected, photographed, and weighed.

### Statistical analysis

All experiments were performed at least three times independently. Data are presented as mean ± standard error of the mean (SEM). Quantitative data were compared between groups using the Student’s t-test (two-tailed, two-sample unequal variance). Statistical significance was set at *p* < 0.05.

## Results

### Cytoplasmic translocation of CIC-S mediated by nuclear ERK

We evaluated the effect of EGF treatment on the stability and nucleocytoplasmic distribution of CIC in HEK293T cells. Total CIC levels were not dramatically altered until 6 h after EGF treatment (Supplementary Figure S1A). In contrast, nuclear CIC-S levels significantly decreased within 2 h of EGF treatment, accompanied by an increase in cytoplasmic CIC-S levels (Supplementary Figure S1B). Thereafter, the nuclear and cytoplasmic CIC-S levels recovered to pre-EGF treatment levels (Supplementary Figure S1B). CIC-L was predominantly present in the nucleus (Supplementary Figure S1B), which is consistent with previous findings (Chittaranjan et al., 2014; Wang et al., 2017). Notably, the cytoplasmic translocation of CIC-L upon EGF treatment was not observed (Supplementary Figure S1B). These results indicate that in our experimental conditions, EGF treatment mainly affected the nucleocytoplasmic distribution of CIC-S and not the stability of CIC in HEK293T cells.

**FIGURE 1:**
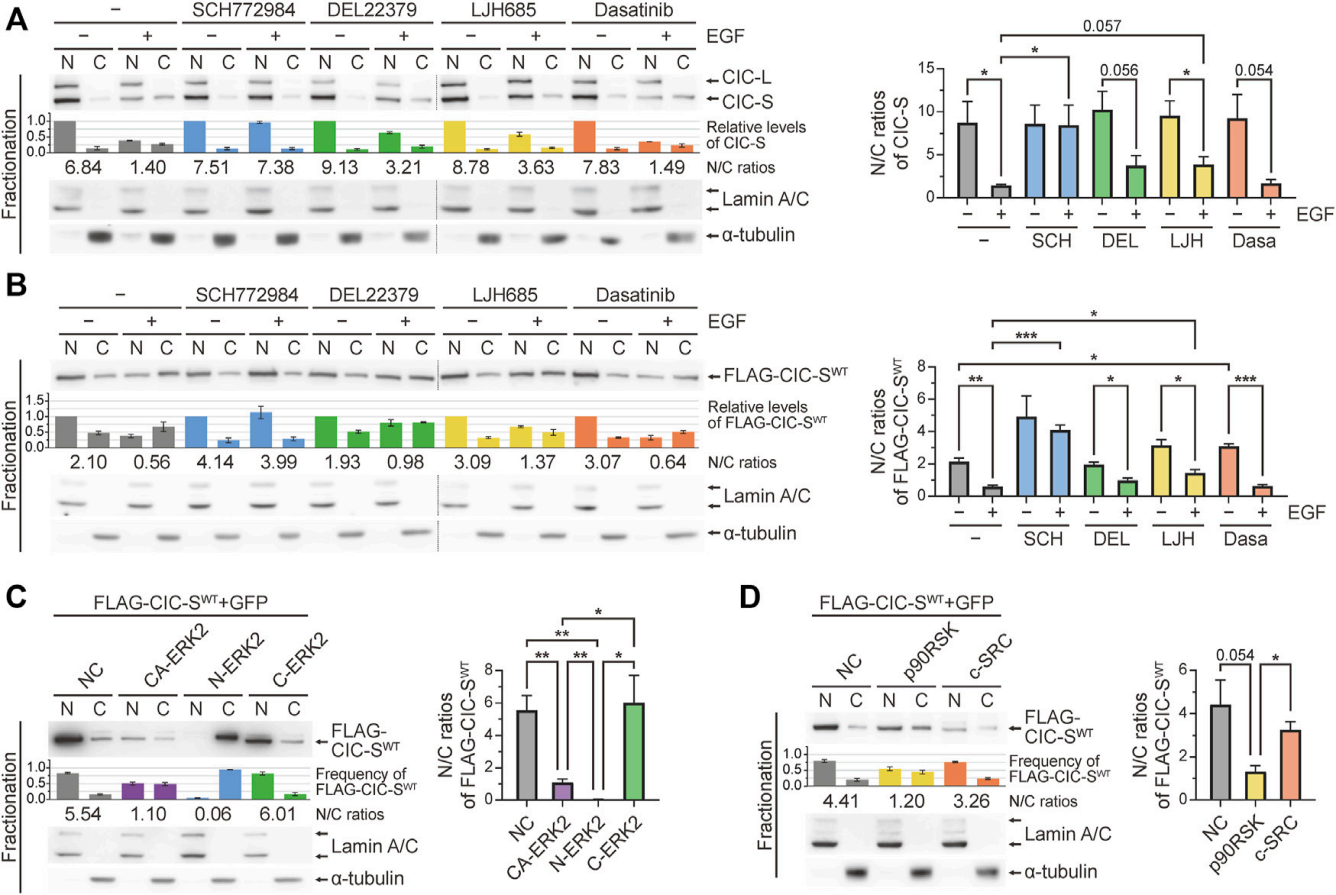
Cytoplasmic translocation of CIC-S mediated by ERK activation. **(A)** Western blotting was performed to determine the effect of kinase inhibitors on changes in the subcellular distribution of CIC in HEK293T cells upon EGF treatment for 30 min. The combination of separate blot images is indicated by dotted lines. The bar graph below the CIC blot image shows the relative levels of CIC-S that were not statistically analyzed. The numbers below the bar graph indicate the N/C ratios of CIC-S. The right panel is a bar graph of the N/C ratios of CIC-S with statistical analysis. Three independent experiments were performed. Error bars indicate SEM. *p < 0.05. SCH772984: ERK1/2 inhibitor, DEL22379: ERK dimerization inhibitor, LJH685: p90RSK inhibitor, and dasatinib: c-SRC inhibitor. **(B)** Western blotting was performed to determine the effect of kinase inhibitors on changes in the subcellular distribution of exogenous mouse CIC-S (FLAG-CIC-S^WT^) in HEK293T cells upon EGF treatment for 30 min. The combination of separate blot images is indicated by dotted lines. The bar graph below the FLAG-CIC-S^WT^ blot image shows the relative levels of FLAG-CIC-S^WT^ that were not statistically analyzed. The numbers below the bar graph indicate the N/C ratios of FLAG-CIC-S^WT^. The right panel is a bar graph of the N/C ratios of FLAG-CIC-S^WT^ with statistical analysis. Three independent experiments were performed. Error bars indicate SEM. *p < 0.05, **p < 0.01, and ***p < 0.001. **(C)** Western blotting was performed to determine the effect of overexpression of constitutively active (CA-ERK2), nuclear (N-ERK2), and cytoplasmic (C-ERK2) ERK2 on the cytoplasmic translocation of FLAG-CIC-S^WT^ in HEK293T cells. The bar graph below the FLAG-CIC-S^WT^ blot image shows the frequency of FLAG-CIC-S^WT^ in the nucleus and cytoplasm, which were not statistically analyzed. The numbers below the bar graph indicate the N/C ratios of FLAG-CIC-S^WT^. The right panel is a bar graph of the N/C ratios of FLAG-CIC-S^WT^ with statistical analysis. Three independent experiments were performed. Error bars indicate SEM. *p < 0.05 and **p < 0.01. **(D)** Western blotting was performed to determine the effect of overexpression of p90RSK and c-SRC on the cytoplasmic translocation of FLAG-CIC-S^WT^ in HEK293T cells. The bar graph below the FLAG-CIC-S^WT^ blot image shows the frequency of FLAG-CIC-S^WT^ in the nucleus and cytoplasm, which were not statistically analyzed. The numbers below the bar graph indicate the N/C ratios of FLAG-CIC-S^WT^. The right panel is a bar graph of the N/C ratios of FLAG-CIC-S^WT^ with statistical analysis. Three independent experiments were performed. Error bars indicate SEM. *p < 0.05. N: nuclear fraction and C: cytoplasmic fraction. N/C: nuclear-to-cytoplasmic ratio.

Three different kinases have been reported to mediate the phosphorylation and cytoplasmic translocation of CIC-S induced by RTK activation: ERK (Futran et al., 2015; Wang et al., 2017), p90RSK (Dissanayake et al., 2011; Ren et al., 2020), and c-SRC (Bunda et al., 2020). We investigated the role of these three kinases in the cytoplasmic translocation of CIC-S by treating HEK293T cells with EGF and their respective kinase inhibitors. Among the kinase inhibitors tested, SCH772984 (ERK1 and ERK2 inhibitor) showed the strongest suppression of EGF treatment-induced cytoplasmic translocation of CIC-S (Figure 1A). LJH685 (p90RSK inhibitor) less efficiently inhibited the cytoplasmic translocation of CIC-S than did SCH772984, whereas dasatinib (c-SRC inhibitor) had a negligible effect (Figure 1A). Notably, DEL22379, an ERK dimerization inhibitor (Herrero et al., 2015), blocked the cytoplasmic translocation of CIC-S to a lower extent than that mediated by SCH772984 (Figure 1A). Total CIC levels were largely unaffected by treatment with the kinase inhibitors (Supplementary Figure S2A). We also examined the effect of kinase inhibitors on cytoplasmic translocation of exogenous FLAG-tagged wild-type mouse CIC-S (FLAG-CIC-S^WT^) in HEK293T cells. As observed for endogenous CIC-S, the kinase inhibitors exerted similar effects on the FLAG-CIC-S^WT^ (Figure 1B; Supplementary Figure S2B). Treatment with MG132, a proteasome complex inhibitor, did not increase FLAG-CIC-S^WT^ levels in both the nucleus and cytoplasm of EGF-treated HEK293T cells (Supplementary Figure S1C), which was consistent with the results of no significant decrease in CIC levels upon EGF treatment (Supplementary Figures S2A, B). This result ruled out the possibility of proteasomal degradation of CIC-S by EGF treatment-induced activation of nuclear ERK (Bunda et al., 2019) in our experimental setting.

Adenosine triphosphate-competitive inhibition of ERK1 and ERK2 by SCH772984 almost completely blocked the cytoplasmic translocation of CIC-S, whereas DEL22379-induced inhibition of ERK dimerization had only a marginal effect (Figures 1A, B). Since ERK dimers interact with and activate cognate cytoplasmic substrates (Casar et al., 2008), we hypothesized that nuclear ERK may be mainly involved in the cytoplasmic translocation of CIC-S. To test this hypothesis, we determined the amount of FLAG-CIC-S^WT^ translocated into the cytoplasm upon overexpression of constitutively active forms of ERK2 (CA-ERK2), nuclear ERK2 (Myc-ERK2-MEK1-LA; N-ERK2), and cytoplasmic ERK2 (Myc-ERK2-MEK1; C-ERK2) (Robinson et al., 1998). CA-ERK2 overexpression induced the cytoplasmic translocation of FLAG-CIC-S^WT^ (Figure 1C; Supplementary Figure S2C). Notably, this phenomenon was induced by the overexpression of N-ERK2 but not by the overexpression of C-ERK2 (Figure 1C; Supplementary Figure S2C), suggesting that nuclear ERK mediates cytoplasmic translocation of CIC-S. We also determined the effects of overexpression of p90RSK and c-SRC on the cytoplasmic translocation of FLAG-CIC-S^WT^. p90RSK overexpression had a stronger effect on the cytoplasmic translocation of FLAG-CIC-S^WT^ than that mediated by c-SRC overexpression (Figure 1D; Supplementary Figure S2D), which was consistent with the results of the kinase inhibitor treatment experiments (Figures 1A,B). Collectively, our results demonstrate that nuclear ERK is a key kinase that mediates cytoplasmic translocation of CIC-S in mammals.

### Identification of serine and threonine residues critical for the cytoplasmic translocation of CIC-S upon phosphorylation

**FIGURE 2:**
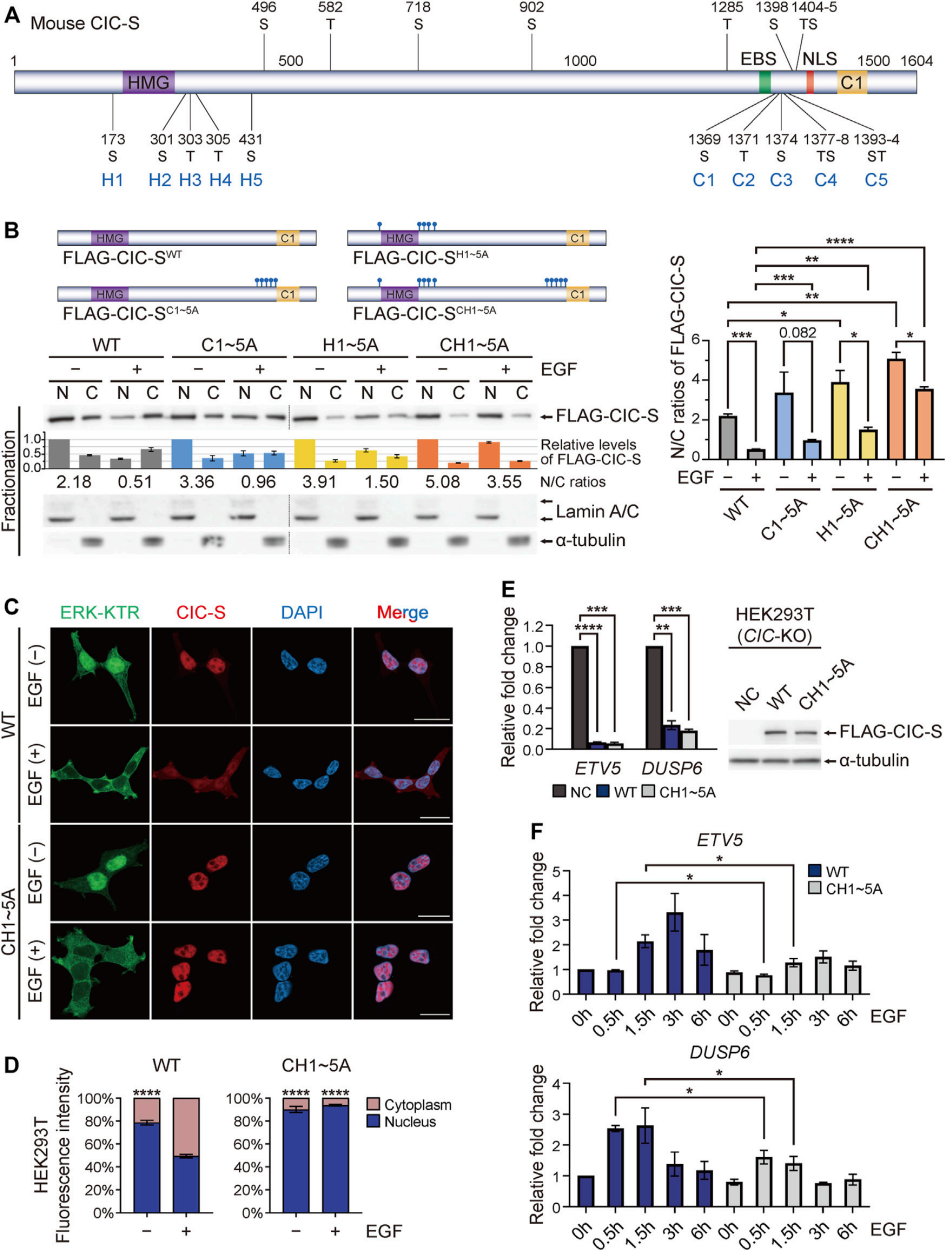
Identification of serine and threonine residues in CIC contributing to EGF treatment-induced cytoplasmic translocation of CIC-S. **(A)** Schematic of domains and potential EGF treatment-induced phosphorylation sites in mouse CIC-S. Serine (S) and threonine (T) residues substituted with alanine are denoted by C1 to C5 and H1 to H5. EBS: ERK-binding site. NLS: nuclear localization signal. **(B)** Western blotting was performed to examine changes in the subcellular distribution of FLAG-CIC-S^WT^, FLAG-CIC-S^C1∼5A^, FLAG-CIC-S^H1∼5A^, and FLAG-CIC-S^CH1∼5A^ in HEK293T cells upon EGF treatment for 30 min. The upper panel shows the schematics of FLAG-CIC-S^WT^, FLAG-CIC-S^C1∼5A^, FLAG-CIC-S^H1∼5A^, and FLAG-CIC-S^CH1∼5A^. Combination of separate blot images is indicated by dotted lines. The bar graph below the FLAG-CIC-S blot image shows the relative levels of FLAG-CIC-S that were not statistically analyzed. The numbers below the bar graph indicate the N/C ratios of FLAG-CIC-S. The right panel is a bar graph of the N/C ratios of FLAG-CIC-S with statistical analysis. Three independent experiments were performed. Error bars indicate SEM. *p < 0.05, **p < 0.01, ***p < 0.001, and ****p < 0.0001. N: nuclear fraction and C: cytoplasmic fraction. N/C: nuclear-to-cytoplasmic ratio. **(C)** Immunofluorescence staining of FLAG-CIC-S^WT^ and FLAG-CIC-S^CH1∼5A^ in HEK293T cells stably expressing ERK-kinase translocation reporter (ERK-KTR). Cells were treated with EGF for 30 min. Green fluorescence indicates that ERK-KTR migrates to the cytoplasm when ERK is activated. All scale bars are 20 μm. **(D)** Quantitative analysis of the percentage of FLAG-CIC-S fluorescence intensity in the nucleus and cytoplasm of individual HEK293T cells with and without EGF treatment. More than 4 cells per group were analyzed. Error bars indicate SEM. ****p < 0.0001. **(E)** qRT-PCR analysis for the expression levels of *ETV5* and *DUSP6* in *CIC-*KO HEK293T cells stably expressing either FLAG-CIC-S^WT^ or FLAG-CIC-S^CH1∼5A^. Western blot images show the levels of FLAG-CIC-S^WT^ and FLAG-CIC-S^CH1∼5A^ in the corresponding stable cell lines. Three independent experiments were performed. The bar graph presents data as the mean ± SEM values. **p < 0.01, ***p < 0.001, and ****p < 0.0001. **(F)** qRT-PCR analysis for time-dependent changes in the expression levels of *ETV5* and *DUSP6* in *CIC-*KO HEK293T cells stably expressing either FLAG-CIC-S^WT^ or FLAG-CIC-S^CH1∼5A^ after treatment with EGF. Three independent experiments were performed. The bar graph presents data as mean ± SEM values. *p < 0.05.

p90RSK is a downstream kinase of ERK and induces 14-3-3-mediated cytoplasmic translocation of CIC *via* phosphorylation of the S173 and S301 residues of human CIC-S (Ren et al., 2020). We verified that alanine substitution of these two serine resides (FLAG-CIC-S^H12A^) significantly blocked the EGF treatment-induced cytoplasmic translocation of FLAG-CIC-S (Supplementary Figure S3A). ERK inhibition further inhibited the cytoplasmic translocation of FLAG-CIC-S^H12A^ upon EGF treatment (Supplementary Figure S3B), which was consistent with the finding that ERK inhibition had a greater suppressive effect on this process than that of p90RSK (Figures 1A,B). Based on these results, we hypothesized that there are more ERK-mediated phosphorylation sites within CIC, which contribute to the EGF treatment-induced cytoplasmic translocation of CIC-S. EGF treatment induces phosphorylation of human CIC-S at 20 different serine and threonine (S/T) residues (Dissanayake et al., 2011). The corresponding S/T residues in mouse CIC-S are shown in Figure 2A. Notably, among the 20 S/T residues, 11 residues are located near the ERK-binding site of CIC-S (Figure 2A). Deletion of the C-terminal region of CIC-S from 1,302 to 1,397 amino acid residues markedly suppressed the EGF treatment-induced nuclear export of CIC-S (Supplementary Figure S3C), suggesting that seven S/T residues (C1–C5) within the deleted region may be critical for the EGF treatment-induced cytoplasmic translocation of CIC-S. Alanine substitution for the seven S/T residues (FLAG-CIC-S^C1∼5A^) markedly inhibited the cytoplasmic translocation of FLAG-CIC-S upon EGF treatment (Figure 2B). We also determined the role of the S/T residues near the HMG box (H1–H5) in the cytoplasmic translocation of CIC-S. Alanine substitution for T303, T305, and S431 residues (FLAG-CIC-S^H345A^) did not significantly inhibit the cytoplasmic translocation of FLAG-CIC-S upon EGF treatment (Supplementary Figure S3A). However, combined alanine substitution for the five S/T residues near the HMG box (FLAG-CIC-S^H1∼5A^) strongly inhibited the EGF treatment-induced cytoplasmic translocation of FLAG-CIC-S to a greater extent than that mediated by substitution for S173 and S301 (Figure 2B; Supplementary Figure S3A). Finally, we simultaneously substituted the seven and five S/T residues near the C1 domain and HMG-box, respectively, with alanine residues (FLAG-CIC-S^CH1∼5A^). This mutation most dramatically blocked the cytoplasmic translocation of FLAG-CIC-S upon EGF treatment (Figure 2B). Immunofluorescence staining verified that cytoplasmic translocation of FLAG-CIC-S^CH1∼5A^ was not induced by EGF treatment (Figures 2C, D).

Since FLAG-CIC-S^CH1∼5A^ contained point mutations at 12 amino acid residues, we examined whether FLAG-CIC-S^CH1∼5A^ still retained its transcriptional repressor activity. To precisely monitor the transcriptional repressor activity of FLAG-CIC-S in cells, we generated *CIC-*knockout (KO) HEK293T cells using the clustered regularly interspaced short palindromic repeats (CRISPR)-Cas9 system (Supplementary Figure S4). Overexpression of FLAG-CIC-S^WT^ and FLAG-CIC-S^CH1∼5A^ dramatically suppressed the expression of CIC target genes, including *ETV5* and dual specificity phosphatase 6 (*DUSP6*), in *CIC-*KO HEK293T cells with similar efficiency (Figure 2E). We also analyzed the time-dependent changes in *ETV5* and *DUSP6* expression levels after EGF treatment. After 30–90 min, the expression of *ETV5* and *DUSP6* was derepressed in FLAG-CIC-S^WT^-expressing *CIC-*KO HEK293T cells, whereas the suppression of *ETV5* and *DUSP6* expression was maintained in FLAG-CIC-S^CH1∼5A^-expressing *CIC-*KO HEK293T cells (Figure 2F). *ETV5* and *DUSP6* were downregulated again in FLAG-CIC-S^WT^-expressing *CIC-*KO HEK293T cells 6 h after EGF treatment (Figure 2F), which was consistent with the results of recovery of nuclear CIC-S levels 4–5 h after EGF treatment (Supplementary Figure S1B). Collectively, these data indicated that EGFR activation induces phosphorylation at S/T residues near the C1 domain and HMG box of CIC-S, leading to cytoplasmic translocation of CIC-S followed by derepression of CIC target gene expression.

### EGF treatment disrupted the interaction between the N-terminal and C-terminal regions of CIC-S and promoted their cytoplasmic translocation

**FIGURE 3:**
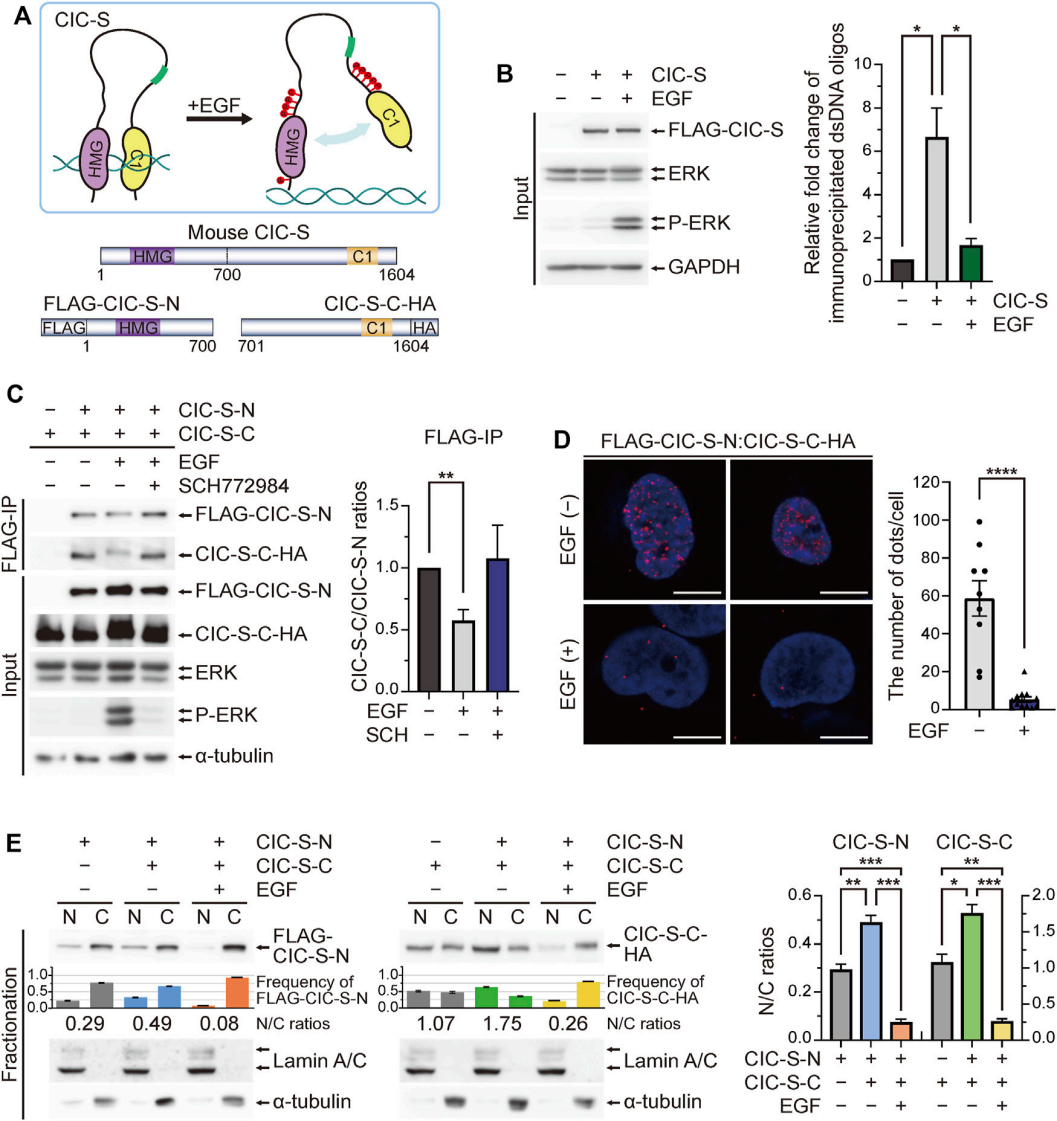
Interaction between the N-terminal and C-terminal regions of CIC for nuclear localization. **(A)** The upper panel shows a schematic model for the disruption of intramolecular interaction between the N-terminal and C-terminal regions of CIC upon phosphorylation. The lower panel shows schematics of FLAG-CIC-S-N and CIC-S-C-HA. **(B)** *In vitro* DNA binding assay. HEK293T cells transfected with control or FLAG-CIC-S^WT^-expressing plasmids were treated with either PBS or EGF for 5 min. Cell extract was incubated with double stranded DNA (dsDNA) oligo composed of six CIC binding motifs for 1 h, followed by immunoprecipitation of FLAG-CIC-S^WT^ using FLAG antibody-conjugated beads. The amount of dsDNA bound to FLAG-CIC-S^WT^ was analyzed by qPCR. The bar graph shows the relative levels of immunoprecipitated dsDNA oligos in each group. Three independent experiments were performed. Error bars indicate SEM. Western blot images for the levels of FLAG-CIC-S^WT^ in HEK293T cells treated with either PBS or EGF are presented in the left panel. *p < 0.05. **(C)** Co-immunoprecipitation of FLAG-CIC-S-N and CIC-S-C-HA. HEK293T cells co-transfected with plasmids expressing FLAG-CIC-S-N and CIC-S-C-HA were treated with EGF and ERK inhibitor (SCH772984), followed by paraformaldehyde fixation and immunoprecipitation using an anti-FLAG antibody. The bar graph shows the ratio of immunoprecipitated CIC-S-C-HA/FLAG-CIC-S-N. Three independent experiments were performed. Error bars indicate SEM. **p < 0.01. **(D)** Proximity ligation assay for the interaction between FLAG-CIC-S-N and CIC-S-C-HA. HEK293T cells co-transfected with plasmids expressing FLAG-CIC-S-N and CIC-S-C-HA were treated with phosphate-buffered saline (PBS) or EGF for 5 min. The bar graph shows the average number of dots representing the interaction between FLAG-CIC-S-N and CIC-S-C-HA in each cell. More than nine cells per group were analyzed. All scale bars are 10 μm. Error bars indicate SEM. ****p < 0.0001. **(E)** Western blotting was performed to analyze the subcellular distribution of FLAG-CIC-S-N and CIC-S-C-HA in HEK293T cells when they were expressed simultaneously or separately. The co-transfected HEK293T cells were treated with PBS or EGF for 5 min. The bar graphs below the blot images show the frequency of FLAG-CIC-S-N and CIC-S-C-HA in the nucleus and cytoplasm. The numbers below the bar graph indicate the N/C ratios of FLAG-CIC-S-N and CIC-S-C-HA. The bar graphs of the N/C ratios of FLAG-CIC-S-N and CIC-S-C-HA with statistical analysis are presented in the right panel. Three independent experiments were performed. Error bars indicate SEM. *p < 0.05, **p < 0.01, and ***p < 0.001. N: nuclear fraction and C: cytoplasmic fraction. N/C: nuclear-to-cytoplasmic ratio.

CIC can recognize specific octameric DNA sequences *via* intramolecular interactions between the HMG box and the C1 domain (Fores et al., 2017) (Figure 3A). CIC target genes are derepressed by RTK-ERK activation even before nuclear export of CIC in *Drosophila* (Lim et al., 2013; Keenan et al., 2020). Based on this knowledge, we hypothesized that EGF treatment will interfere with the intramolecular interaction of CIC, thus promoting dissociation from DNA and cytoplasmic translocation of CIC-S (Figure 3A). To test our hypothesis, we first examined whether EGF treatment decreases DNA binding affinity of CIC-S *in vitro*. HEK293T cells were transfected with plasmids expressing FLAG-CIC-S^WT^ and treated with either PBS or EGF. Cell extract was incubated with dsDNA oligo composed of six CIC binding motifs, followed by immunoprecipitation of FLAG-CIC-S^WT^ using FLAG antibody-conjugated beads. DNA bound to FLAG-CIC-S^WT^ was analyzed by qPCR. The amount of DNA bound to FLAG-CIC-S^WT^ was significantly reduced upon EGF treatment (Figure 3B), indicating that EGF treatment indeed decreased DNA binding activity of CIC-S.

Next, we constructed plasmids expressing either the N-terminal region (FLAG-CIC-S-N) or the carboxyl (C)-terminal region (CIC-S-C-HA) of CIC-S (Figure 3A) and co-transfected HEK293T cells with the plasmids followed by either cross-linking and immunoprecipitation assays using an anti-FLAG antibody or proximity ligation assays using anti-FLAG and anti-HA antibodies. FLAG-CIC-S-N and CIC-S-C-HA interacted with each other (Figures 3C, D). The interaction between FLAG-CIC-S-N and CIC-S-C-HA was predominantly found in the nucleus (Figure 3D). EGF treatment decreased the interaction between FLAG-CIC-S-N and CIC-S-C-HA (Figures 3C, D), supporting our hypothesis. Moreover, ERK inhibition restored the interaction between FLAG-CIC-S-N and CIC-S-C-HA in the presence of EGF (Figure 3C), demonstrating that EGF treatment-induced ERK activation disrupts the intramolecular interaction of CIC-S.

Finally, we analyzed the subcellular localization of FLAG-CIC-S-N and CIC-S-C-HA. When FLAG-CIC-S-N and CIC-S-C-HA were expressed simultaneously, the N/C ratio of FLAG-CIC-S-N and CIC-S-C-HA was higher than when they were expressed separately (Figure 3E), indicating the interaction between the N-terminal and C-terminal regions of CIC-S for facilitating nuclear localization. However, EGF treatment induced the cytoplasmic translocation of FLAG-CIC-S-N and CIC-S-C-HA even when they were co-expressed (Figure 3E). Collectively, our findings suggest that ERK activation disrupts the intramolecular interaction between the N-terminal and C-terminal regions of CIC-S, leading to dissociation from DNA and cytoplasmic translocation of CIC-S.

### Inhibition of cytoplasmic translocation of CIC-S suppressed tumor growth

**FIGURE 4:**
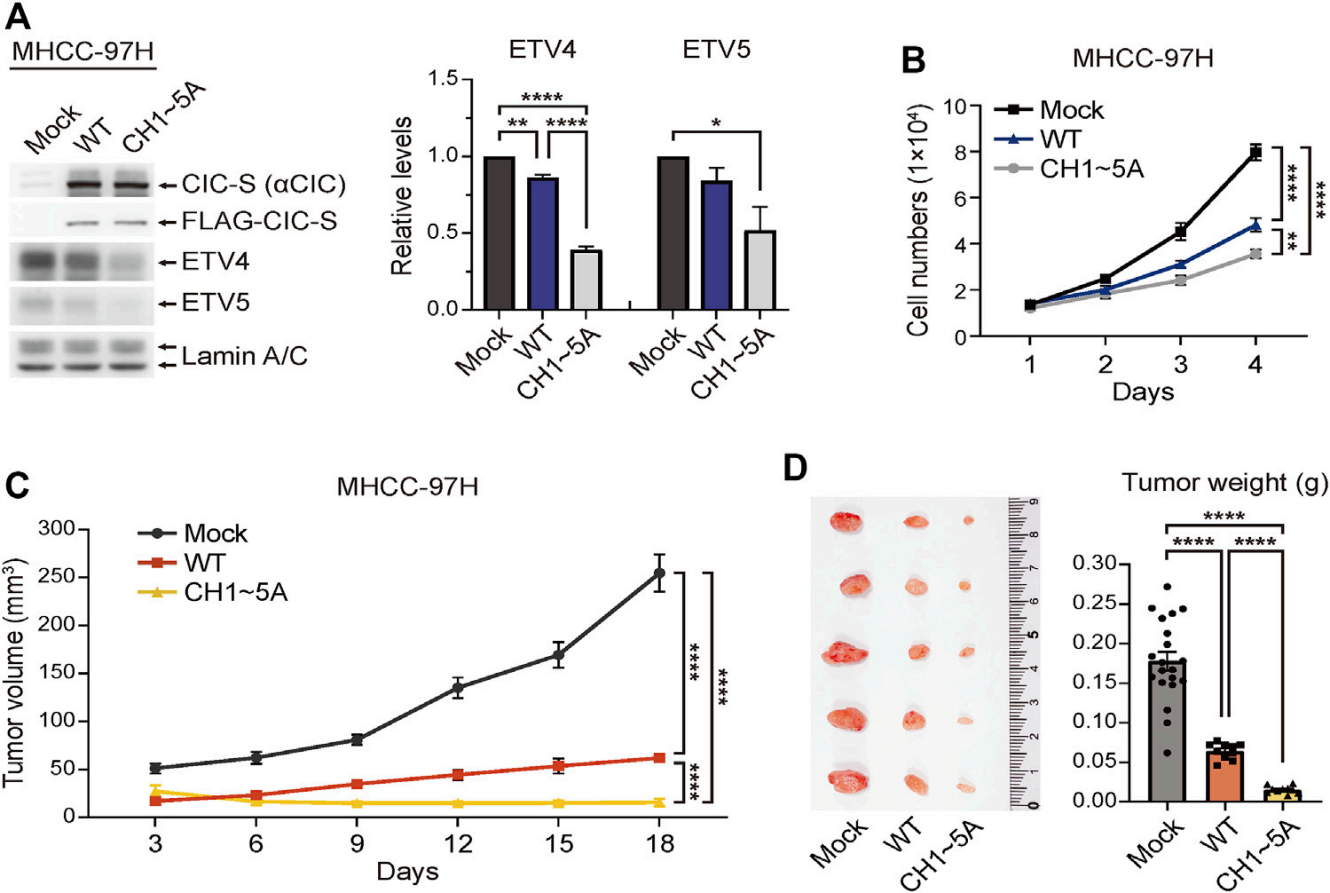
Enhanced tumor suppressor activity of FLAG-CIC-S^CH1∼5A^. **(A)** Western blotting was performed to determine the expression levels of FLAG-CIC-S, ETV4, and ETV5 in MHCC-97H cells stably expressing FLAG-CIC-S^WT^ or FLAG-CIC-S^CH1∼5A^. The right panel is a bar graph for the relative level of ETV4 and ETV5. Three independent experiments were performed. Error bars indicate SEM. *p < 0.05, **p < 0.01, and ****p < 0.0001. **(B)** *In vitro* cell growth assay for mock, FLAG-CIC-S^WT^-, and FLAG-CIC-S^CH1∼5A^-expressing MHCC-97H cell lines. Cells were counted using a hemacytometer every 24 h for 4 days. Error bars represent SEM. *n* = 8 per group. **p < 0.01 and ****p < 0.0001. **(C,D)** *In vivo* tumor growth assay using xenograft mouse models. Tumor **(C)** growth curves and **(D)** weights of nude mice inoculated with mock, FLAG-CIC-S^WT^-, and FLAG-CIC-S^CH1∼5A^-expressing MHCC-97H cells. Tumor volumes were measured every 3 days for 18 days. On day 18, tumors were collected, photographed, and weighed. Error bars represent SEM. The bar graph presents data as mean ± SEM values. ****p < 0.0001.

CIC functions as a tumor suppressor in various cancers, including hepatocellular carcinoma (HCC) (Kim et al., 2018; Lee, 2020). Although many studies have determined the effect of regulating CIC expression on cancer progression, the effect of modulating cytoplasmic translocation of CIC on tumor growth has never been investigated. To examine the effect of blocking the cytoplasmic translocation of CIC-S on cancer cell growth, we generated MHCC-97H HCC cell lines stably overexpressing either FLAG-CIC-S^WT^ or FLAG-CIC-S^CH1∼5A^ (Figure 4A). The overexpression of FLAG-CIC-S^CH1∼5A^ strongly suppressed the expression of ETV4 and ETV5 proteins, which are direct CIC targets and well-known oncogenic transcription factors (Oh et al., 2012), to a greater extent than that mediated by FLAG-CIC-S^WT^ in MHCC-97H cells (Figure 4A). Consistent with this result, cell proliferation was dramatically decreased by the overexpression of FLAG-CIC-S^CH1∼5A^ to a greater extent than that observed upon FLAG-CIC-S^WT^ overexpression in MHCC-97H cells (Figure 4B). We verified these results *in vivo* using xenograft mouse models. Normal and FLAG-CIC-S^WT^- or FLAG-CIC-S^CH1∼5A^-overexpressing MHCC-97H cells were subcutaneously injected into either posterior flank of the same nude mice, respectively, and the tumor volume was measured every 3 days. FLAG-CIC-S^CH1∼5A^-overexpressing MHCC-97H cells showed slower growth and formed a smaller tumor mass than those of FLAG-CIC-S^WT^-overexpressing MHCC-97H cells *in vivo* (Figures 4C, D). ETV4 expression was dramatically suppressed in tumors derived from FLAG-CIC-S^CH1∼5A^-overexpressing MHCC-97H cells to a greater extent than in those derived from FLAG-CIC-S^WT^-overexpressing MHCC-97H cells when compared to that in normal MHCC-97H-derived tumors (Supplementary Figure S5). These results indicate that nuclear retention of CIC inhibits tumor growth *via* suppression of CIC target genes involved in promoting cancer progression.

## Discussion

**FIGURE 5:**
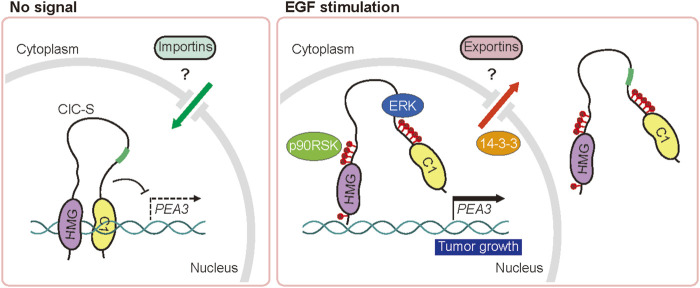
Schematic model of the regulation of nucleocytoplasmic transport of CIC by the EGFR signaling pathway. EGFR stimulation-activated ERK and p90RSK disrupt the intramolecular interaction of CIC *via* phosphorylation of serine and threonine residues (red circles), leading to dissociation from DNA and cytoplasmic translocation of CIC-S.

In this study, we showed that nuclear ERK plays a key role in the cytoplasmic translocation of CIC-S in mammals. It is believed that ERK-activated p90RSK phosphorylates CIC-S at the S173 and S301 residues to induce the 14-3-3-mediated inhibition of DNA binding and/or nuclear export of CIC-S (Dissanayake et al., 2011; Ren et al., 2020). Our study confirmed the importance of the S173 and S301 residues in the cytoplasmic translocation of CIC-S and further identified additional S/T residues near the C1 domain and HMG box of CIC-S that contribute to this process when phosphorylated by ERK activation. C1–C4 S/T residues near the C1 domain follow the ERK phosphorylation motif, which is S or T residue followed by a proline residue ((pS/T)P motif), whereas C5 and H5 S residues fit the p90RSK phosphorylation motif (RXXpS). The mutagenesis experiment clearly revealed that accumulation of alanine substitution of the S/T residues progressively inhibited the cytoplasmic translocation of CIC-S upon EGF treatment (Figure 2B; Supplementary Figure S3A). Moreover, EGF treatment disrupted the interaction between the N-terminal and C-terminal regions of CIC-S and promoted cytoplasmic translocation of CIC-S. These data suggest that phosphorylation of the S173 and S301 residues along with other S/T residues near the C1 domain and HMG box of CIC-S results in a conformational change in CIC-S, and consequently dissociates CIC from DNA and recruits protein factors required for nuclear export of CIC-S including 14-3-3 (Figure 5). Future studies should perform structural analysis of CIC-S according to phosphorylation status and identify the exportins and importins involved in nucleocytoplasmic transport of CIC-S to elucidate the regulation of CIC activity in mammals.

The present study could not verify the previous finding that c-SRC mediates the EGF treatment-induced cytoplasmic translocation of CIC-S *via* phosphorylation of Y1455 residue in human CIC-S (Bunda et al., 2020). c-SRC inhibitor treatment did not suppress the cytoplasmic translocation of CIC-S in HEK293T cells upon EGF treatment (Figures 1A,B). Moreover, EGF treatment still induced the cytoplasmic translocation of FLAG-CIC-S containing a phenylalanine substitution at Y1451 residue (FLAG-CIC-S^Y1451F^), corresponding to Y1455 of human CIC-S, in our experimental setting (Supplementary Figure S6). However, in the absence of EGF, the N/C ratio of FLAG-CIC-S^Y1451F^ was slightly higher than that of FLAG-CIC-S^WT^ (Supplementary Figure S6), and c-SRC overexpression decreased the N/C ratio of FLAG-CIC-S^WT^ (Figure 1D), although those were not statistically significant. Therefore, c-SRC phosphorylation of the Y1451 residue may contribute to the cytoplasmic translocation of CIC-S independent of the EGFR signaling pathway.

Enhancing the activity of tumor suppressors can inhibit cancer progression (Xue et al., 2007). CIC activity can be regulated *via* proteasomal degradation and/or cytoplasmic translocation (Astigarraga et al., 2007; Ajuria et al., 2011; Okimoto et al., 2017; Lee, 2020). Our study demonstrated that blocking the cytoplasmic translocation of CIC-S potently suppressed tumor growth (Figures 4C, D). Notably, the expression level of FLAG-CIC-S^CH1∼5A^ was higher than that of FLAG-CIC-S^WT^ in MHCC-97H-derived tumors (Supplementary Figure S5), while their expression levels were similar between each MHCC-97H cell line used in the xenograft experiments (Figure 4A). These results suggest that inhibition of cytoplasmic translocation may increase the stability and transcriptional repressor activity of CIC-S during tumor formation. Since CIC functions as a tumor suppressor in various cancers, elucidating the regulatory mechanisms of nucleocytoplasmic transport of CIC will help develop therapeutic strategies applicable to various cancers.

## Data Availability

The original contributions presented in the study are included in the article/Supplementary Material, further inquiries can be directed to the corresponding author.
