# Supplementary material for: ERK phosphorylation disrupts the intramolecular interaction of capicua to promote cytoplasmic translocation of capicua and tumor growth
Source: Front Mol Biosci. 2022 Dec 22;9:1030725. doi: 10.3389/fmolb.2022.1030725 (PMC9814488; doi:10.3389/fmolb.2022.1030725)
Supplement: Supplementary file 1 [file DataSheet2.PDF]

Fig S2

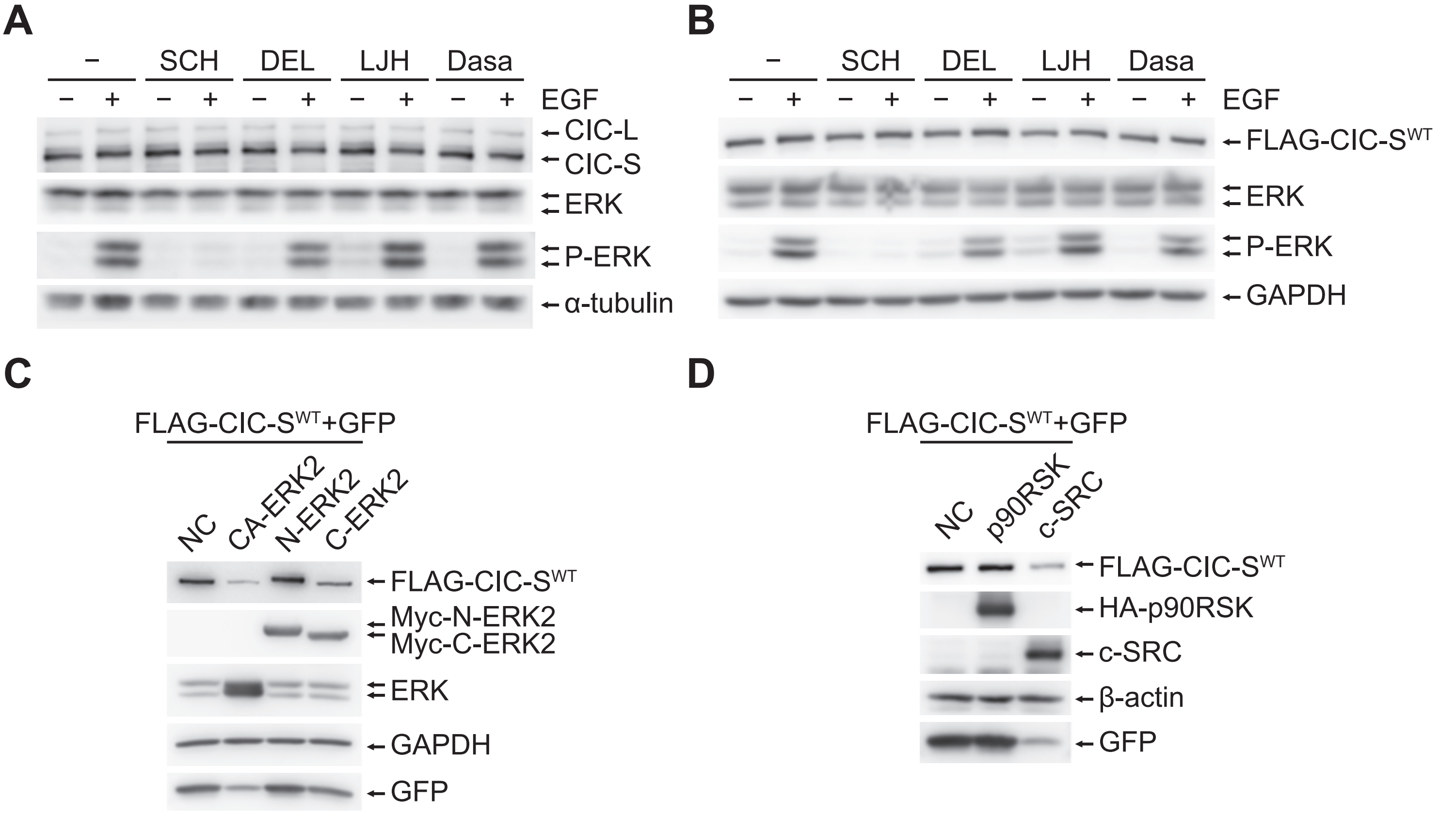

Supplementary Figure S2. Western blotting of total protein levels for Figure 1. (A and B) Western blotting of total protein levels for Figures. 1A **(A)** and 1B **(B)**. SCH (SCH772984): ERK1/2 inhibitor, DEL (DEL22379): ERK dimerization inhibitor, LJH (LJH685): p90RSK inhibitor, and Dasa (dasatinib): c-SRC inhibitor. **(C and D)** Western blotting of total protein levels for Figures. 1C **(C)** and 1D **(D)**. GFP was used as a control for transfection efficiency. CA-ERK2: constitutively active ERK2, N-ERK2: nuclear ERK2 (Myc-ERK2-MEK1-LA), and C-ERK2: cytoplasmic ERK2 (Myc-ERK2-MEK1).
