# Supplementary material for: ERK phosphorylation disrupts the intramolecular interaction of capicua to promote cytoplasmic translocation of capicua and tumor growth
Source: Front Mol Biosci. 2022 Dec 22;9:1030725. doi: 10.3389/fmolb.2022.1030725 (PMC9814488; doi:10.3389/fmolb.2022.1030725)
Supplement: Supplementary file 2 [file DataSheet4.PDF]

Fig S4

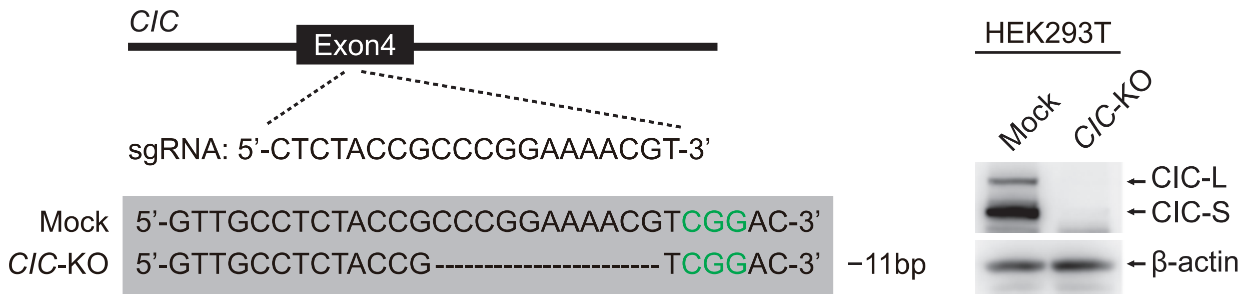

Supplementary Figure S4. Generation of CIC-KO HEK293T cells using the CRISPR-Cas9 system. The left panel shows the deleted DNA sequences within exon 4 of CIC in CIC-KO HEK293T cells. Western blot images in the right panel show no CIC expression in CIC-KO HEK293T cells.
