# Supplementary material for: ERK phosphorylation disrupts the intramolecular interaction of capicua to promote cytoplasmic translocation of capicua and tumor growth
Source: Front Mol Biosci. 2022 Dec 22;9:1030725. doi: 10.3389/fmolb.2022.1030725 (PMC9814488; doi:10.3389/fmolb.2022.1030725)
Supplement: Supplementary file 3 [file DataSheet6.PDF]

Fig S6

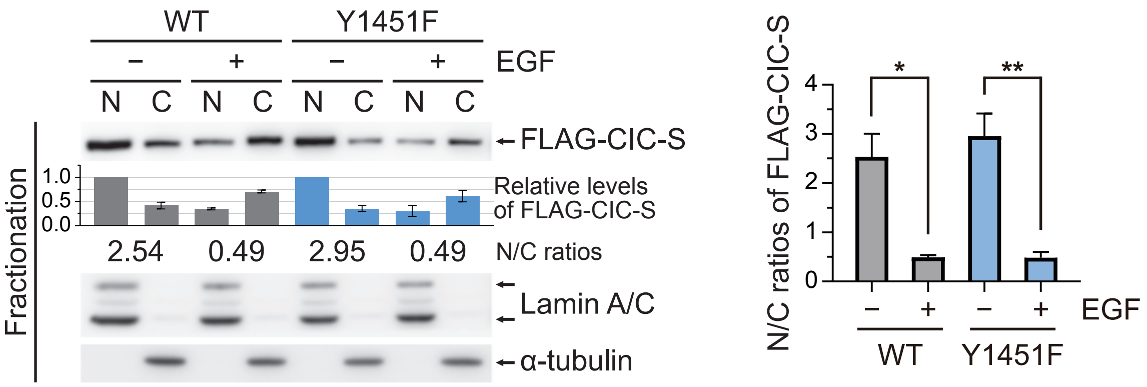

Supplementary Figure S6. Cytoplasmic translocation of FLAG-CIC-S^{Y1451F} upon EGF treatment. Western blotting was performed to examine changes in the subcellular distribution of FLAG-CIC-S^{WT} and FLAG-CIC-S^{Y1451F} in HEK293T cells upon EGF treatment for 30 min. The bar graph below the FLAG-CIC-S blot image shows the relative levels of FLAG-CIC-S that were not statistically analyzed. The N/C ratios of FLAG-CIC-S are shown below the bar graph. The right panel is a bar graph with statistical analysis for the N/C ratios of FLAG-CIC-S. Three independent experiments were performed. Error bars indicate SEM. *$P < 0.05$ and **$P < 0.01$. N: nuclear fraction and C: cytoplasmic fraction. N/C: nuclear-to-cytoplasmic ratio.
