# Supplementary material for: ERK phosphorylation disrupts the intramolecular interaction of capicua to promote cytoplasmic translocation of capicua and tumor growth
Source: Front Mol Biosci. 2022 Dec 22;9:1030725. doi: 10.3389/fmolb.2022.1030725 (PMC9814488; doi:10.3389/fmolb.2022.1030725)
Supplement: Supplementary file 4 [file DataSheet3.PDF]

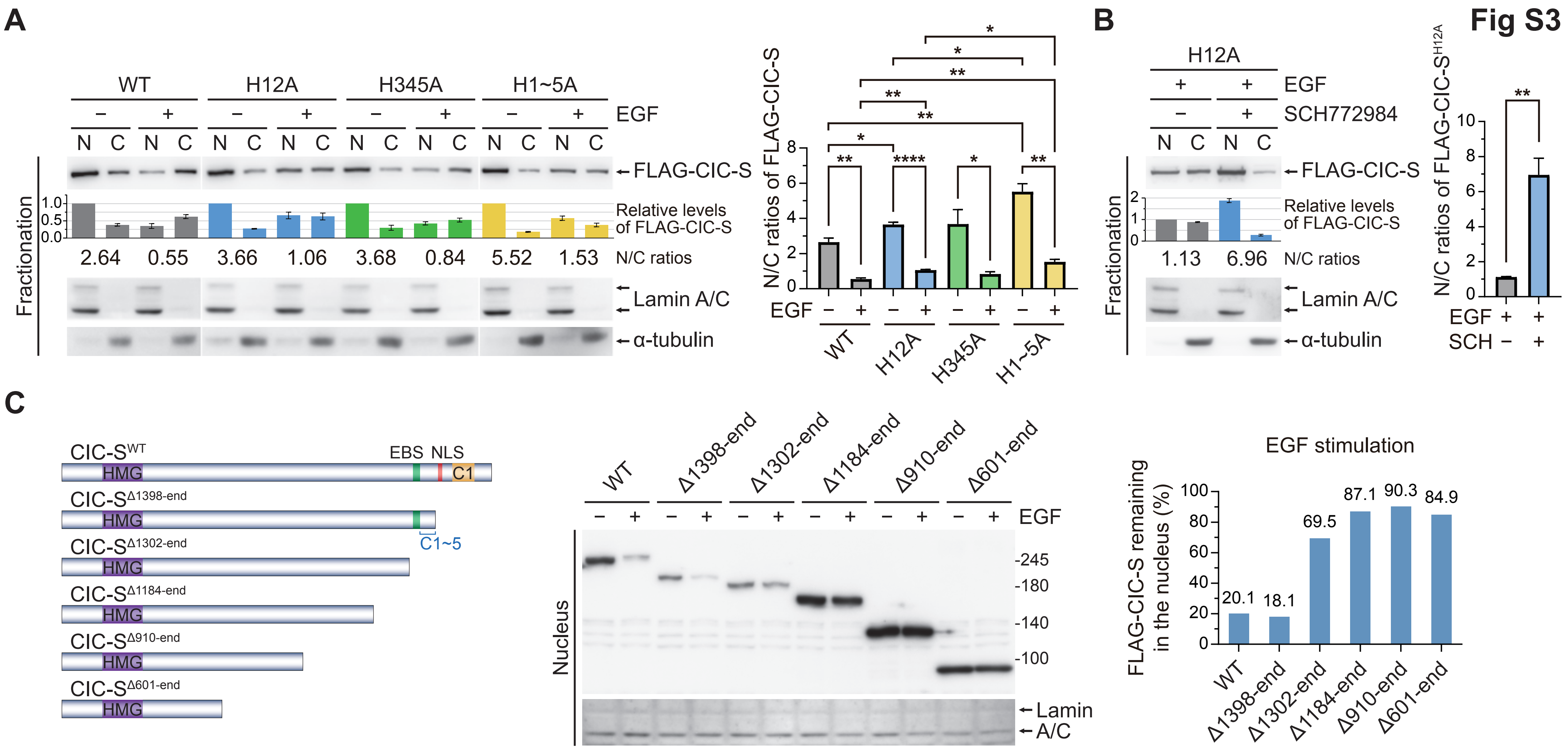

Supplementary Figure S3. Mutagenesis to identify serine and threonine residues in CIC-S that contribute to the cytoplasmic translocation of CIC-S upon EGF treatment. (A) Western blotting was performed to examine changes in the subcellular distribution of FLAG-CIC-S^{WT}, FLAG-CIC-S^{H12A}, FLAG-CIC-S^{H345A}, and FLAG-CIC-S$^{H1\sim5A}$ in HEK293T cells upon EGF treatment for 30 min. The bar graph below the FLAG-CIC-S blot image shows the relative levels of FLAG-CIC-S that were not statistically analyzed. The N/C ratios of FLAG-CIC-S are shown below the bar graph. The right panel is a bar graph with statistical analysis for the N/C ratios of FLAG-CIC-S. Three independent experiments were performed. Error bars indicate SEM. *$P < 0.05$, **$P < 0.01$, and ****$P < 0.0001$. N: nuclear fraction and C: cytoplasmic fraction. N/C: nuclear-to-cytoplasmic ratio. **(B)** Western blotting was performed to determine the effect of ERK inhibitor (SCH772984) on changes in the subcellular distribution of FLAG-CIC-S^{H12A} in HEK293T cells upon EGF treatment for 30 min. The bar graph below the FLAG-CIC-S^{H12A} blot image shows the relative levels of FLAG-CIC-S^{H12A} that were not statistically analyzed. The N/C ratios of FLAG-CIC-S^{H12A} are shown below the bar graph. The right panel is a bar graph with statistical analysis for the N/C ratios of FLAG-CIC-S^{H12A}. Three independent experiments were performed. Error bars indicate SEM. **$P < 0.01$. **(C)** Western blotting was performed to determine changes in the levels of WT and a series of C-terminally truncated FLAG-CIC-S in the nucleus of HEK293T cells upon EGF treatment. The bar graph shows the proportion of WT and mutant FLAG-CIC-S remaining in the nucleus 30 min after EGF treatment. Schematics of WT and a series of C-terminally truncated FLAG-CIC-S are presented in the left panel.
