# Supplementary material for: ERK phosphorylation disrupts the intramolecular interaction of capicua to promote cytoplasmic translocation of capicua and tumor growth
Source: Front Mol Biosci. 2022 Dec 22;9:1030725. doi: 10.3389/fmolb.2022.1030725 (PMC9814488; doi:10.3389/fmolb.2022.1030725)
Supplement: Supplementary file 5 [file DataSheet1.PDF]

Fig S1

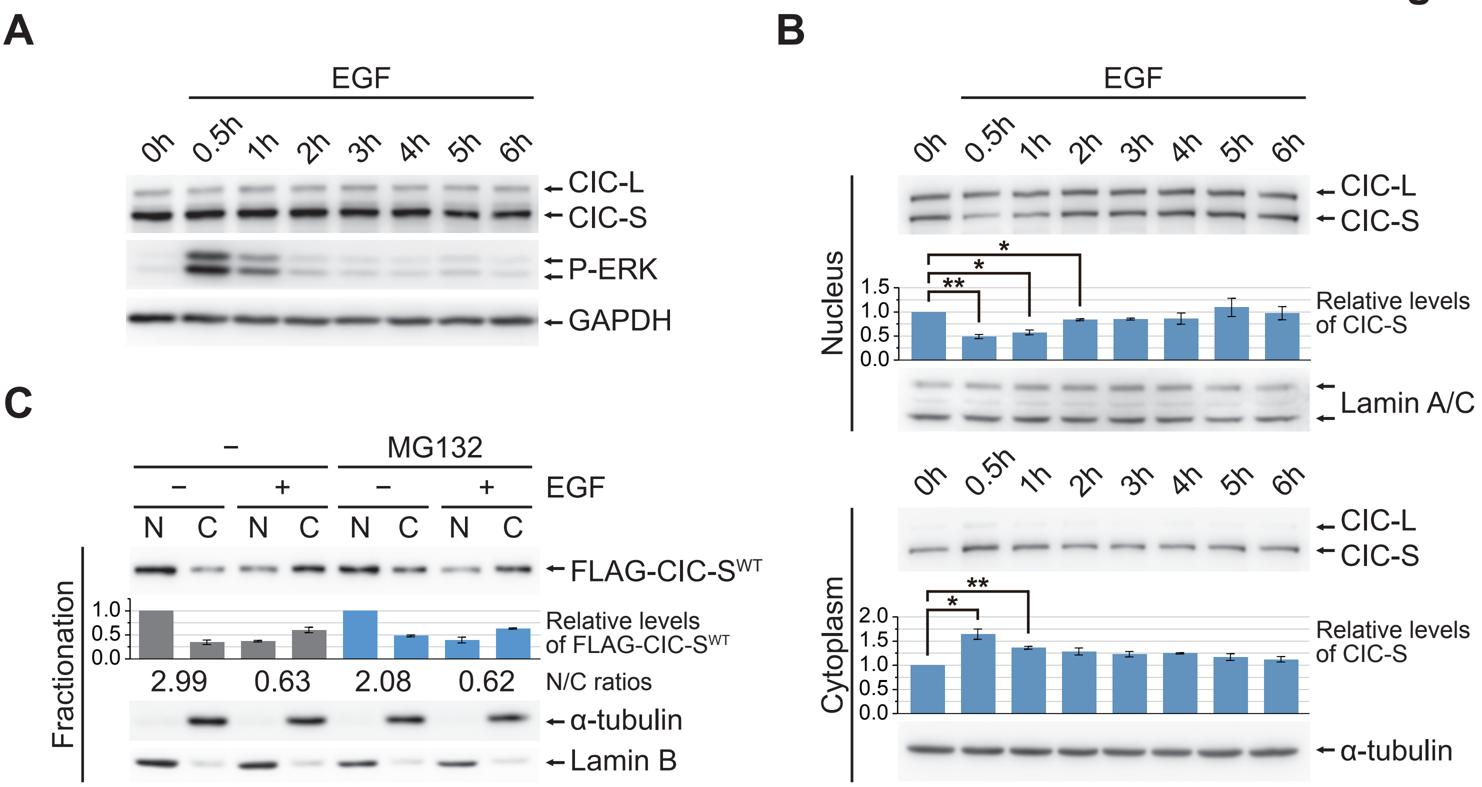

Supplementary Figure S1. Effect of EGF treatment on nucleocytoplasmic distribution and stability of CIC in HEK293T cells. (A and B) Western blotting was performed to investigate time-dependent changes in the levels of **(A)** total CIC and **(B)** nuclear and cytoplasmic CIC in HEK293T cells upon EGF treatment. The bar graphs below the CIC blot images show the relative levels of CIC-S. Three independent experiments were performed. Error bars indicate SEM. *P < 0.05 and **P < 0.01. **(C)** Western blotting was performed to determine the effect of MG132 proteasome inhibitors on nuclear and cytoplasmic FLAG-CIC-S^{WT} levels in HEK293T cells treated with PBS or EGF. The bar graph below the FLAG-CIC-S^{WT} blot image shows the relative levels of FLAG-CIC-S^{WT}. Three independent experiments were performed. The numbers below the bar graph indicate the N/C ratios of FLAG-CIC-S^{WT}. N: nuclear fraction and C: cytoplasmic fraction. N/C: nuclear-to-cytoplasmic ratio.
