# Supplementary material for: ERK phosphorylation disrupts the intramolecular interaction of capicua to promote cytoplasmic translocation of capicua and tumor growth
Source: Front Mol Biosci. 2022 Dec 22;9:1030725. doi: 10.3389/fmolb.2022.1030725 (PMC9814488; doi:10.3389/fmolb.2022.1030725)
Supplement: Supplementary file 6 [file DataSheet5.PDF]

Fig S5

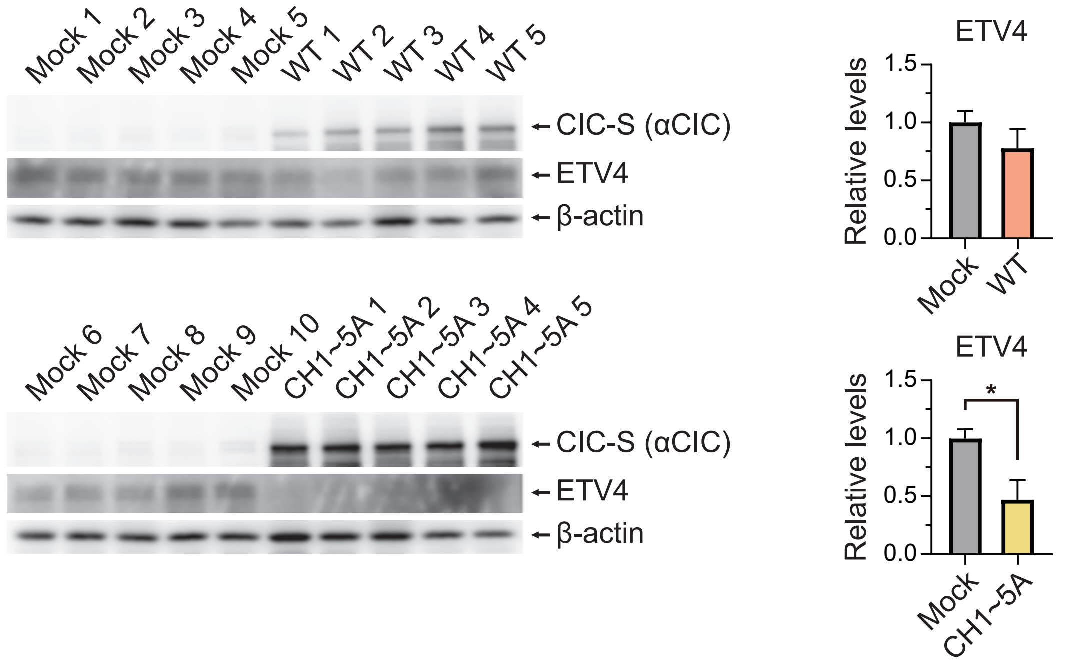

Supplementary Figure S5. Comparison of FLAG-CIC-S and ETV4 levels in MHCC-97H-derived tumors. Western blotting was performed to examine the levels of ETV4 and CIC-S in tumors derived from mock, FLAG-CIC-S^{WT}-, and FLAG-CIC-S$^{\mathrm{CH1\sim5A}}$-expressing MHCC-97H cell lines. CIC-S levels were determined using an anti-CIC antibody (αCIC). Two western blot images for CIC-S levels were obtained at the same exposure time. Five individual tumor samples per group were subjected to western blotting. The bar graphs show the relative levels of ETV4. Error bars indicate SEM. *$P < 0.05$.
